# Lnc RNA ZFAS1 regulates the proliferation, apoptosis, inflammatory response and autophagy of fibroblast-like synoviocytes via miR-2682-5p/ADAMTS9 axis in rheumatoid arthritis

**DOI:** 10.1042/BSR20201273

**Published:** 2020-08-18

**Authors:** Shanshan Yang, Wei Yin, Yan Ding, Fan Liu

**Affiliations:** Department of Immunology and Rheumatology, Wuhan Children’s Hospital (Wuhan Maternal and Child Healthcare Hospital), Tongji Medical College, Huazhong University of Science and Technology, WuHan, China

**Keywords:** cell autophagy, inflammatory, Rheumatoid arthritis

## Abstract

**Backgrounds:** Rheumatoid arthritis (RA) is a frequent autoimmune disease. Emerging evidence indicated that ZNFX1 antisense RNA1 (ZFAS1) participates in the physiological and pathological processes in RA. However, knowledge of ZFAS1 in RA is limited, the potential work pathway of ZFAS1 needs to be further investigated.

**Methods:** Levels of ZFAS1, microRNA (miR)-2682-5p, and ADAM metallopeptidase with thrombospondin type 1 motif 9 (ADAMTS9) were estimated using quantitative real-time polymerase chain reaction (qRT-PCR) assay. 3-(4,5-Dimethylthiazol-2-yl)-2,5-diphenyltetrazolium bromide (MTT) assay was conducted to explore the ability of cell proliferation in fibroblast-like synoviocytes (FLS-RA). Cell apoptosis was measured via flow cytometry. Also, levels of ADAMTS9, apoptosis-related proteins, cleaved-caspase-3 (active large subunit), and autophagy-related proteins were identified adopting Western blot. Enzyme-linked immunosorbent assay (ELISA) was performed to determine the productions of inflammatory cytokines. Beside, the interrelation between miR-2682-5p and ZFAS1 or ADAMTS9 was verified utilizing dual-luciferase reporter assay.

**Results:** High levels of ZFAS1 and ADAMTS9, and a low level of miR-2682-5p were observed in RA synovial tissues and FLS-RA. Knockdown of ZFAS1 led to the curbs of cell proliferation, inflammation, autophagy, and boost apoptosis in FLS-RA, while these effects were abolished via regaining miR-2682-5p inhibition. Additionally, the influence of miR-2682-5p on cell phenotypes and inflammatory response were eliminated by ADAMTS9 up-regulation in FLS-RA. Mechanically, ZFAS1 exerted its role through miR-2682-5p/ADAMTS9 axis in RA.

**Conclusion:** ZFAS1/miR-2682-5p/ADAMTS9 axis could modulate the cell behaviors, inflammatory response in FLS-RA, might provide a potential therapeutic target for RA treatment.

## Introduction

Rheumatoid arthritis (RA) is one of the most common chronic joint diseases characterized by the aggressive proliferation of synoviocytes, accompanied by the secretions of inflammatory cytokines and chemokines [1]. A series of records have revealed that RA is strictly related to the genetic combination [2,3]. Currently, RA has seriously reduced the quality of life in RA patients who account for approximately 1% of the population around the world [4]. Emerging investigations manifest that cytokines act vital functions in the development of RA, such as interleukin (IL)-6 and tumor necrosis factor (TNF)-α [5,6]. However, the therapeutic effect is unsatisfactory, other diagnosed and treated biomarkers need to be highlighted.

Long non-coding RNAs (lncRNAs) have attracted mountainous attention for their association with the homeostasis and biological modulation, such as cell growth, apoptosis, inflammatory response, and immunity [7]. By definition, lncRNAs consists of more than 200 nucleotides in length, and there is increasing evidence that lncRNAs can be used for numerous inflammatory diseases, including RA [8,9]. For example, metastasis-associated lung adenocarcinoma transcript 1 (MALAT1) could regulate cellular apoptosis of fibroblast-like synoviocytes (FLSs) in RA [10]. LncRNA ZNFX1 antisense RNA1 (ZFAS1) was a cancer-related lncRNA [11,12], it contributed to oncogenesis via sponging microRNA (miRNA/miR)-150-5p in melanoma [13]. Besides, ZFAS1 also could trigger the mobility of hepatocellular carcinoma [14]. Based on the features of ZFAS1 in cell behaviors, we considered whether ZFAS1 could participate in the progression of RA.

As we all know, lncRNAs act as the sponge of miRNAs that are a class of RNAs with 18–24 nucleotides in length [15]. Over the past decades, miRNAs play considerable roles in pathogenesis and tumorigenesis [16], such as miR-29b acts as a tumor suppressor to curb cell metastasis of prostate cancer [17]. MiR-2682-3p restrains the proliferation of osteosarcoma cells by regulating matrix metalloproteinase 8 and Cyclin D2 [18]. Not only that, miRNAs have been suggested to be the master regulator in autoimmune diseases [19]. For example, miR-146 participated in the production of the pro-inflammatory factor in human gingival fibroblasts [20]. However, miR-2682-5p, as a novel miRNAs, the functional researches and molecular mechanism needed to be further explored. In addition, the regulatory mechanism of miRNAs has been reported to target the mRNAs via sequence complementarity [21]. A disintegrin-like and metalloproteinase domain with thrombospondintype 1 motifs (ADAMTS9) belongs to ADAMTS proteases, reduces the adhesion of cells [22]. However, the function of ADAMTS9 in inflammatory diseases was less researched before. Herein, we aimed to expose whether miR-2682-5p bound to ADAMTS9 in RA pathogenesis.

In the paper, we paid attention to the expression profiles of ZFAS1, miR-2682-5p, and ADAMTS9 in RA synovial tissues and cells, and the network about them was also the objective of the present investigation.

## Materials and methods

### Clinical specimens

For the samples collection, the healthysynovial tissues (normal, *n*=30) were taken out from the patients with trauma, who were undergoing arthroscopic surgery for knee ligament injury. And the RA synovial specimens (RA, *n*=30) were donated from the RA patients who were suffering from total knee arthroplasty. All the participators were treated in Wuhan Maternal and Child Healthcare Hospital. Above all, written informed consents were signed by the participators, and this research was ratified by the Ethical Committee of Wuhan Maternal and Child Healthcare Hospital.

### Isolation and cell culture

We firstly separated the cells from normal and RA samples as per the previous descriptions [23]. Briefly, the tissues were minced into small fragments and then placed in a tube which filled with 2 mg/ml of type II collagenase (Invitrogen, Carlsbad, CA, U.S.A.). After digestion for 2 h, the cells were collected via centrifugationat 210 × ***g*** for 5 min. Next, the cells (marked as FLS-Normal and FLS-RA) were re-suspended with high-glucose Dulbecco’s modified eagle medium (DMEM; Hyclone, Logan, UT, U.S.A.) containing 10% fetal bovine serum (FBS; Gibco, Carlsbad, CA, U.S.A.) 100 U/ml penicillin and 100 μg/ml streptomycin. A humidified atmosphere with 5% CO_2_ was suited to incubate the FLS-RA at 37°C.

### Transient transfection

MiR-2682-5p mimics (miR-2682-5p) and inhibitor (anti-miR-2682-5p), as well as their scrambles (miR-NC and anti-miR-NC) were purchased from GenePharma (Shanghai, China). Small interfering (siRNA) of ZFAS1 (si-ZFAS1#1 and si-ZFAS#2), and its negative control (si-NC) were designed and synthesized in GenePharma. Besides, the full-length of ADAMTS9 or mutant sequence was cloned into pcDNA3.1 plasmid to form overexpression vector of ADAMTS9 (ADAMTS9) and its control (pcDNA). Then cell transfection was performed by using Lipofectamine 2000 reagent (Invitrogen) following the producer’s instructions.

### Quantitative real-time polymerase chain reaction (qRT-PCR) assay

Total RNA from synovial tissues and FLS-RA cells were divided and extracted with the help of Trizol Reagent (Invitrogen) in accordance with the guidelines. Reverse transcription was administrated through adopting the PrimeScript RT Enzyme mix kit (Takara, Dalian, China) to generate complementary DNA (cDNA). The created cDNA was reacted with Fast SYBR Green PCR kit (Applied Biosystems, Foster City, CA, U.S.A.) to quantify the relative levels of ZFAS1, miR-2682-5p, and ADAMTS9. Glyceraldehyde-3-phosphate dehydrogenase (GAPDH; for ZFAS1 and ADAMTS9) and U6 (for miR-2682-5p) served as the endogenous references via the 2^−ΔΔCt^ method. The primers were as follows:ZFAS1 (Forward: 5′-CTATTGTCCTGCCCGTTAGAG-3′, Reverse: 5′-GTCAGGAGATCGAAGGTTGTAG-3′); ADAMTS9 (Forward: 5′-CATAATGAACAGGATGGGCCT-3′, Reverse: 5′-TTGACCACATCCAGGGGTTG-3′); GAPDH (Forward: 5′-AACGTGTCAGTGGTGGACCTG-3′, Reverse: 5′-AGTGGGTGTCGCTGTTGAAGT-3′);U6 (Forward: 5′-CTCGCTTCGGCAGCACA-3′, Reverse: 5′-AACGCTTCACGAATTTGCGT-3′), miR-2682-5p primers were purchased from GenePharma.

### 3-(4,5-Dimethylthiazol-2-yl)-2,5-diphenyltetrazolium bromide (MTT) assay

To determine the proliferation of FLS-RA, MTT assay was conducted. Briefly, cells (1 × 10^4^ cells/well) were inoculated into a 96-well plate with complete DMEM. Then, 20 μl MTT reagent (5 mg/ml; Sigma, St. Louis, MO, U.S.A.) was added into per well and then cultured for another 4 h at 37°C. Subsequently, the supernatant was abandoned, and 150 μl dimethyl sulfoxide (DMSO; Sigma) was supplemented to dissolve crystals. The absorbance of the lysate was surveyed adopting a microplate reader at 490 nm.

### Flow cytometry assay

FLS-RA that require to be implemented with apoptosis assay were harvested via centrifugation for 3 min under 1500  × ***g***, and the ice-cold phosphate-buffered solution (PBS; Hyclone) was used to wash the cells. Then, Annexin V-fluorescein isothiocyanate (FITC)/propidium iodide (PI) detection kit (BD Biosciences, SanDiego, CA, U.S.A.) was employed to color cells as per the manuals. In the end point, the apoptotic cells were distinguished and analyzed with the help of flow cytometer (BD Biosciences).

### Western blot assay

The protocol of this Western blot was in the light of the previous description [24]. Briefly, the protein extracts with the sample buffer were denatured for 10 min in boiling water, followed by dissociated on a 10% polyacrylamide gel. The isolated protein were transfected onto the Polyvinylidene Fluoride (PVDF; Millipore, Bedford, MA, U.S.A.), and then maintained with primary antibodies (Abcam, Cambridge, MA, U.S.A.), including CD14 (ab183322, 1:1000), CD44 (ab189524, 1:1000), CD90 (ab225, 1:1000), ADAMTS9 (ab32565, 1:2000), LC3B (ab51520, 1:3000), p62 (ab109012, 1:25000), Cleaved-caspase-3 (active large subunit; ab13847, 1:500), BCL2-Associated X (Bax; ab32503, 1:7000), B-Cell leukemia/lymphoma 2 (Bcl-2; ab32124, 1:1000), β-actin (ab8226, 1:6000), and GAPDH (ab8245, 1:5000), of which β-actin and GAPDH acted as the references. Then. the membranes were incubated with corresponding secondary antibody (Abcam). The bands were visualized using an enhanced chemiluminescent substrate kit (Millipore).

### Enzyme-linked immunosorbent assay (ELISA)

In the assay, the secretions of IL-6, IL-10, and tumor necrosis factor (TNF)-α were quantified utilizing relative kits (Neobioscience, Shenzhen, China) according to the protocol. Briefly, FLS-RA cells were transfected with related sequences. After incubation for 48 h, the levels of IL-6, IL-10, and TNF-α in cell supernatant were examined as per relative specifications. Simultaneously, the standard curve was drawn in the same way.

### Dual-luciferase reporter assay

The binding sites between ZFAS1 and miR-2682-5p were amplified and inserted intopmirGLO (Promega, Madison, WI, U.S.A.) vector, generating wild-type (ZFAS1-WT) reporter. The 3′UTR of ADAMTS9 sequence contained the predicted region with miR-2682-5p was used to construct ADAMTS9-WT vector. Simultaneously, the mutant reporters were also formed with the same way, marked as ZFAS1-MUT and ADAMTS9-MUT. Co-transfection with recombinant reporters (ZFAS1-WT, ZFAS1-MUT, ADAMTS9-WT, or ADAMTS9-MUT) and miRNAs (miR-NC or miR-2682-5p) was performed into FLS-RA using Lipofectamine 2000 (Invitrogen). Dual-LuciferaseReporter Assay System kit (Promega) was applied to determine the luciferase activity at 24 h post-transfection.

### Statistical analysis

All statistical analyses were administrated using SPSS 22.0 and expressed as mean ± standard deviation (SD). Difference between the two groups was assayed utilizing Student’s *t*-test. The comparison among multiple groups was performed by one-way analysis of variance with Tukey test. *P*<0.05 was regarded as significant difference.

## Results

### The level of ZFAS1 was highly expressed in RA synovial tissues and FLS-RA

**Figure 1:**
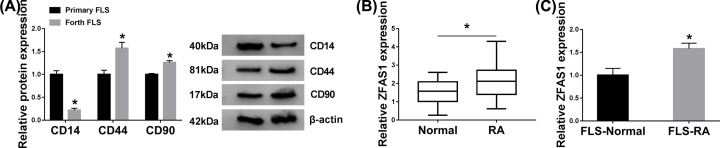
The level of ZFAS1 was highly expressed in RA tissues and FLS-RA (**A**) Protein levels of CD14, CD44, and CD90 were detected in primary FLS and Forth FLS by Western blot assay. (**B** and **C**) QRT-PCR assay was implemented to determine the level of ZFAS1 in RA tissues and FLS-RA in comparison with paired controls; *P<0.05.

First, the primary FLS and Forth FLS was identified by using negative marker CD14, and positive markers (CD44 and CD90). Western blot results suggested that CD14 level was decreased, and CD44 and CD90 were increased in Forth FLS relative to primary FLS, demonstrating the phenotype of Forth FLS. Furthermore, to determine the expression of ZFAS1 in RA patients and healthy people, we measured the level of ZFAS1 in RA synovial tissues compared with matched normal control, qRT-PCR analysis manifested that ZFAS1 was clearly up-regulated in RA group (Figure 1A). Subsequently, FLS-RA and FLS-Normal were segregated from relative synovial tissues, a similar tendency of ZFAS1 level was discovered in FLS-RA (Figure 1B). The data indicated that ZFAS1 was aberrantly expressed in synovial tissues and FLS-RA.

### Knockdown of ZFAS1 decreased proliferation, inflammatory response, autophagy, and increased apoptosis of FLS-RA

**Figure 2:**
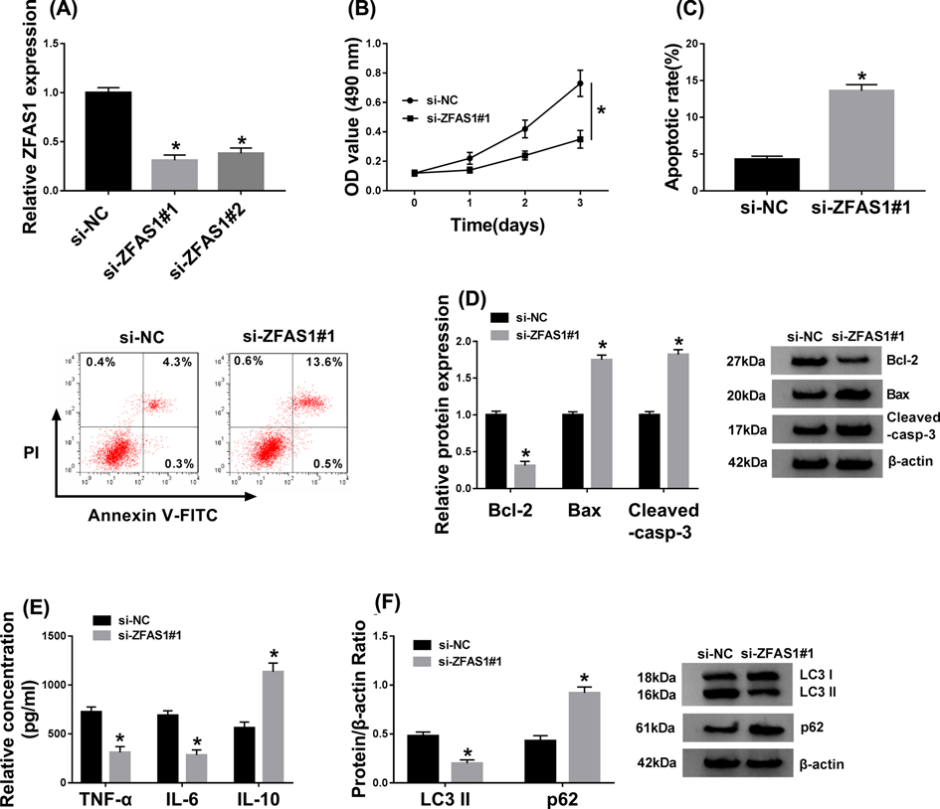
Knockdown of ZFAS1 led to the decreases of cell proliferation, inflammatory response, autophagy, and increase of apoptosis in FLS-RA Si-NC, si-ZFAS1#1, or si-ZFAS1#2 was introduced into FLS-RA, (**A**) and the level of ZFAS1 was examined using qRT-PCR. (**B**) MTT assay was carried out to evaluate cell proliferation in FLS-RA. (**C**) Cell apoptosis was identified via flow cytometry. (**D**) Levels of apoptosis-related proteins (Bcl-2, Bax, and cleaved-casp-3) were estimated by Western blot. (**E**) ELISA was administrated to assess the production of TNF-α, IL-6, and IL-10 *in vitro*. (**F**) Western blot assay was employed to measure the levels of LC3II and p62 in FLS-RA; *P<0.05.

Owing to the ectopic expression of ZFAS1, we presumed that ZFAS1 accounted for the process of RA. First, the knockdown vector of ZFAS1 (si-ZFAS1#1 and si-ZFAS1#2) was constructed introduced into FLS-RA. Both of the knockdown vectors could evidently lessen ZFAS1. Overall consideration, si-ZFAS1#1 was selected for subsequent assays (Figure 2A). Next, we detected the role of ZFAS1 detetion in the proliferation of FLS-RA, MTT analysis presented that ZFAS1 silencing could specially hamper cell proliferation *in vitro* (Figure 2B). Moreover, a significant improvement of cell apoptosis was observed in ZFAS1-silenced FLS-RA (Figure 2C). Also, the effect of ZFAS1 reduction on cell apoptosis was validated via measuring the alterations ofcell apoptosis-related protein levels. The high expression of Bax and cleaved-casp-3 (active large subunit), and the low level of Bcl-2 in si-ZFAS1-transfected FLS-RA implied that ZFAS1 deficiency indeed enhanced cell apoptosis *in vitro* (Figure 2D). Simultaneously, we also identified the influence of ZFAS1 in inflammation, the decreases of TNF-α and IL-6, and the increase of IL-10 suggested that ZFAS1 repression could constrain the inflammatory response in FLS-RA (Figure 2E). Importantly, we also explored the role of ZFAS1 detetion on cell autophagy as per previous descriptions [25]. ZFAS1 silencing particularly suppressed cell autophagy in FLS-RA, showing as the reduction of LC3II and the promotion of p62 (Figure 2F). The evidence meant that ZFAS1 knockdown strikingly weakened cell proliferation, autophagy, inflammatory response, and facilitated cell apoptosis in RA synoviocytes.

### Impact of ZFAS1 silencing on cell behaviors and inflammatory response was abrogated by ADAMTS9 up-regulation *in vitro*

**Figure 3:**
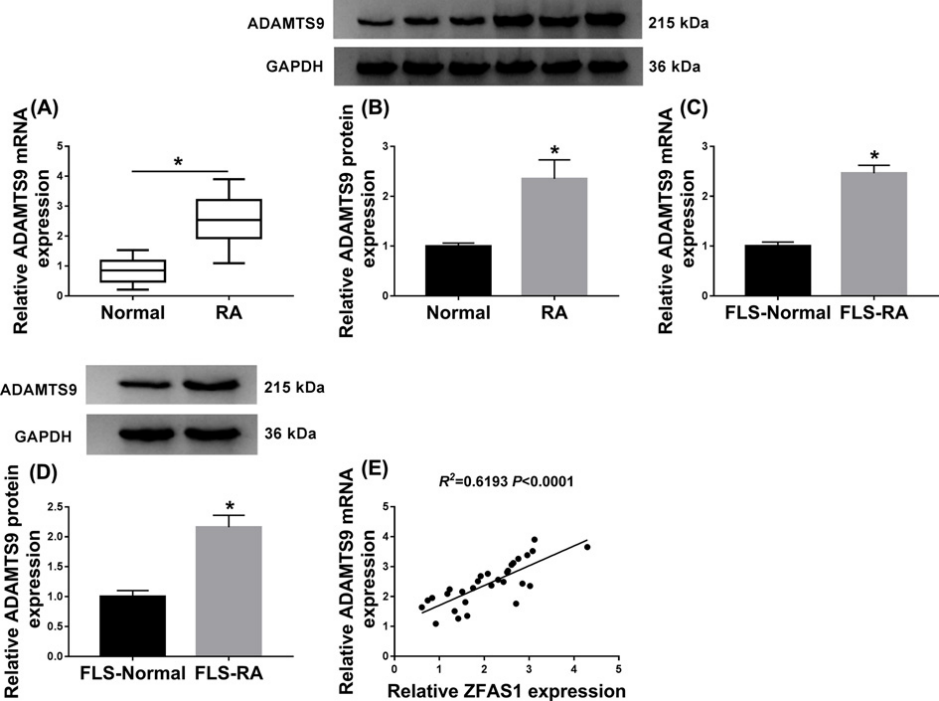
The level of ADAMTS9 was up-regulated in RA synovial tissues and FLS-RA (**A**–**D**) The mRNA and protein levels of ADAMTS9 in (**A** and** C**) RA tissues and (**B** and **D**) FLS-RA were detected using qRT-PCR and Western blot assay, respectively. (**E**) QRT-PCR assay was conducted to analyze the correlation between ADAMTS9 and ZFAS1 in RAsynovial tissues; *P<0.05.

**Figure 4:**
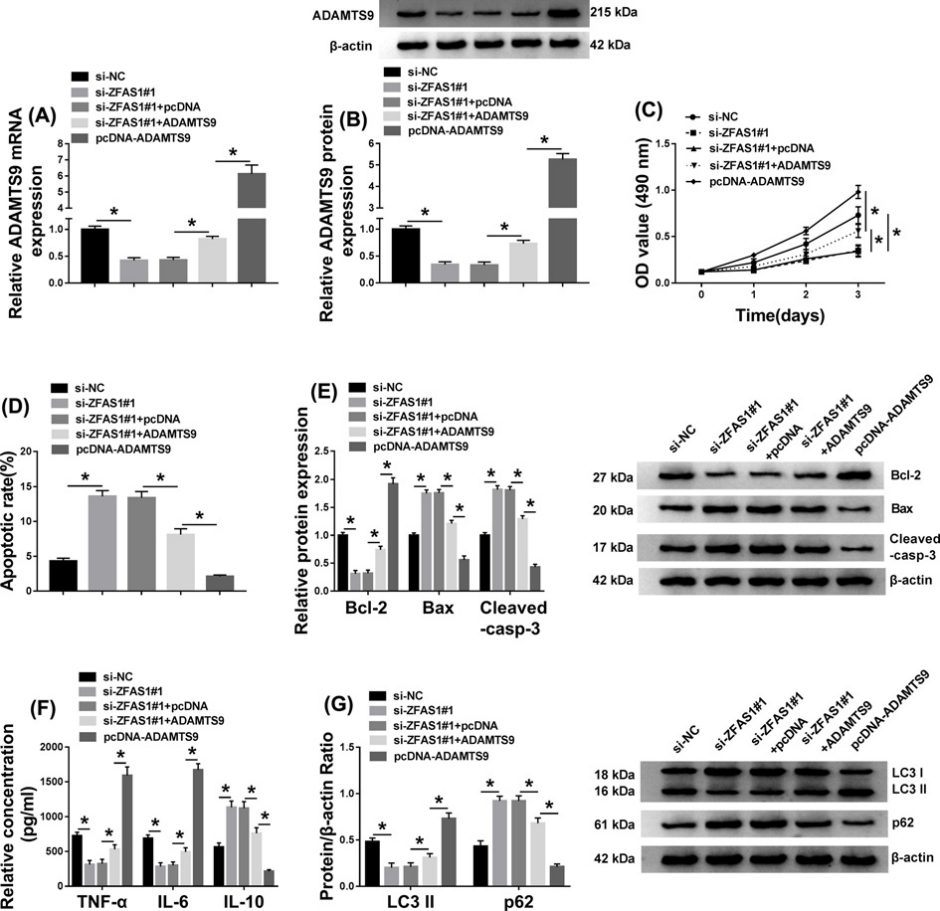
Impact of ZFAS1 silencing on cell behaviors and inflammatory response was abrogated by ADAMTS9 up-regulation* in vitro* FLS-RA were transfected with si-NC, si-ZFAS1#1, si-ZFAS1#1+pcDNA, si-ZFAS1#1+ADAMTS9, or pcDNA-ADAMTS9, respectively. (**A** and **B**) qRT-PCR and Western blot assays were carried out to assess the mRNA and protein levels of ADAMTS9. (**C**) Cell proliferation of FLS-RS was examined utilizing MTT assay. (**D**) The ability of cell apoptosis of FLS-RA was detected adopting flow cytometry. (**E**) Western blot was conducted to measure levels of Bcl-2, Bax, and cleaved-casp-3 in FLS-RA. (**F**) The levels of TNF-α, IL-6, and IL-10 were estimated by ELISA. (**G**) The roles of ZFAS1 detetion and ADAMTS9 overexpression in altering the levels of LC3II and p62 were validated via Western blot; *P<0.05.

According to the previous researches, ADAMTS9 has been revealed to be associated with RA [26], we conjectured whether ZFAS1 could interact with ADAMTS9. First, the high expression of ADAMTS9 in RA synovial tissues at the aspects of mRNA and protein expression was expounded (Figure 3A,B). Besides, a similar expression characters of ADAMTS9 was observed in FLS-RA (Figure 3C,D). Importantly, a positive correlation between ZFAS1 and ADAMTS9 was determined in RA synovial tissues (Figure 3E). This positive correlation between them drove us to further explore their biological functions in RA. Si-ZFAS1#1, si-ZFAS1#1 plus ADAMTS9, or pcDNA-ADAMTS9 was transfected into FLS-RA, and the inhibiting effect of ZFAS1 repression on the mRNA and protein levels of ADAMTS9 was rescued by ADAMTS9 up-regulation *in vitro*, while ADAMTS9 expression level was increased due to the introduction of pcDNA-ADAMTS9 (Figure 4A,B). Afterwards, reintroduction of ADAMTS9 could specially abolish the restraining impact of ZFAS1 detetion on cell proliferation in RA synoviocytes, and the overexpression of ADAMTS9 promoted the proliferative ability of RA synoviocytes (Figure 4C). Meanwhile, cell apoptosis boosted as a result of ZFAS1 silencing, and such promotion was relieved after simultaneous transfection with ADAMTS9 in FLS-RA, whereas apoptosis rate could be hindered caused by the up-regulation of ADAMTS9 (Figure 4D). Also, the alterations of cell apoptosis-related protein levels and cleaved-caspase-3 (active large subunit) illuminated the above conclusion about cell apoptosis *in vitro* (Figure 4E). Synchronously, we assayed the influence of ZFAS1 and ADAMTS9 in inflammation, the hampering role of inflammatory response caused by ZFAS1 reduction was accelerated via co-transfection with ADAMTS9 in RA synoviocytes, and the transfection of pcDNA-ADAMTS9 alone promoted the inflammatory response (Figure 4F). Apart from that, up-regulation of ADAMTS9 also overturned the suppressive impact of ZFAS1 deficiency on the autophagy of FLS-RA, and the introduction of pcDNA-ADAMTS9 alone accelerated cell autophagy (Figure 4G). Namely, the effect of ZFAS1 knockdown on cell behaviors and inflammation was reversed by ADAMTS9 overexpression in FLS-RA.

### ZFAS1 was a sponge of miR-2682-5p to isolate ADAMTS9

**Figure 5:**
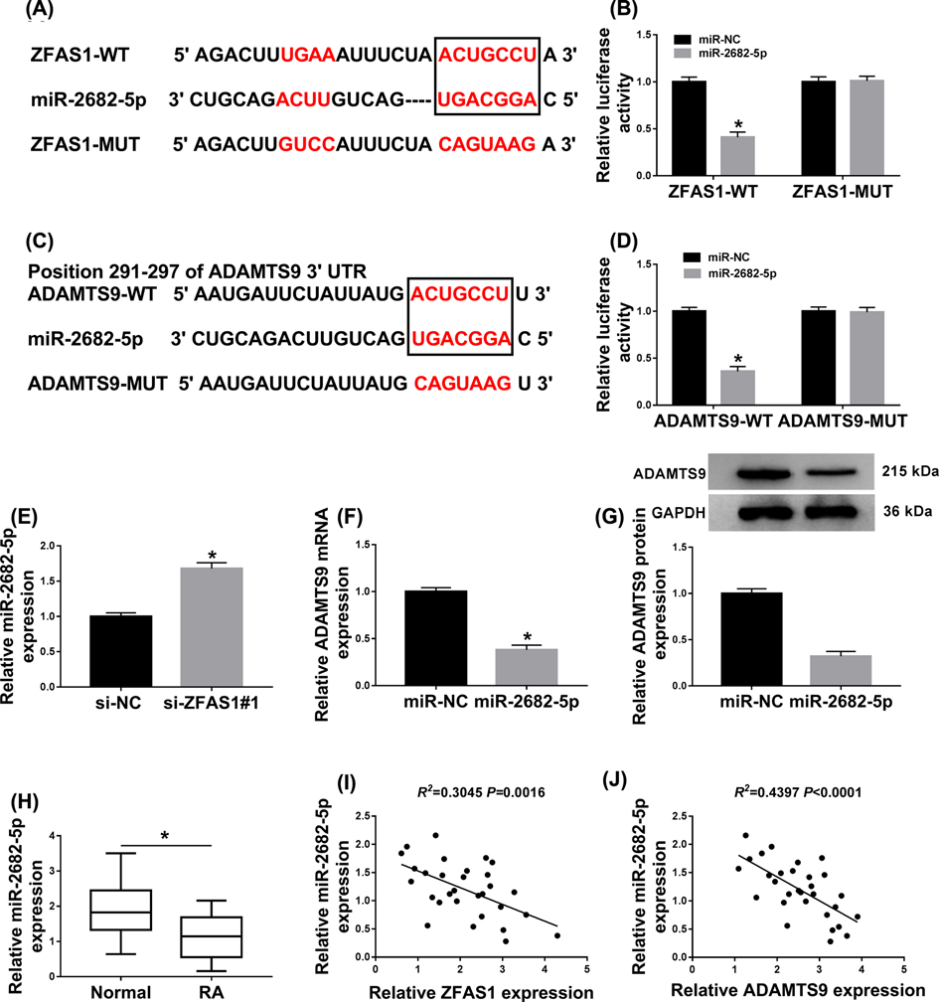
ZFAS1 was a sponge of miR-2682-5p to isolate ADAMTS9 (**A**) The common fragments between ZFAS1 and miR-2682-5p was predicted by starBase v3.0. (**B**) Dual-luciferase reporter assay was used to verify the interrelation between miR-2682-5p and ADAMTS9. (**C**) Predictive binding sites between miR-2682-5p and ADAMTS9 were shown. (**D**) The relationship between miR-2682-5p and ADAMTS9 was confirmed by dual-luciferase reporter assay. (**E**) The level of miR-2682-5p was determined in ZFAS1-silenced FLS-RA by qRT-PCR. (**F** and **G**) The mRNA and protein levels of ADAMTS9 in FLS-RA under miR-2682-5p overexpression. (**H**) QRT-PCR analysis for the level of miR-2682-5p in RA was exhibited. (**I** and** J**) The correlation between miR-2682-5p and ZFAS1 or ADAMTS9 was analyzed; *P<0.05.

In view of the above descriptions, a link between ZFAS1 and ADAMTS9 needed to be explored. As the prediction of starBase v3.0, miR-2682-5p was a probable target of ZFAS1 (Figure 5A). Subsequently, the luciferase reporter (ZFAS1-WT or ZFAS1-MUT) was co-transfected with miR-2682-5p or miR-NC into FLS-RA, the miR-2682-5p overexpression could apparently impede the luciferase activity of ZFAS-WT, but it had no distinct influence on modifying the fluorescence intensity in mutant group (Figure 5B). Interestingly, miRDB predicted that there were complementary sequences between miR-2682-5p and ADAMTS9 (Figure 5C). The reductive role of miR-2682-5p in regulating the luciferase activity of wild-type reporter and the invalid effect of it on mediating the activity of mutant reporter clarified that miR-2682-5p could interact with ADAMTS9 (Figure 5D). Next, an effective augment of miR-2682-5p was observed in ZFAS1-silenced FLS-RA (Figure 5E). Expectantly, miR-2682-5p up-regulation remarkably constrained the level of ADAMTS9 at the aspects of mRNA and protein expression (Figure 5F,G). Also, the decreased expression of miR-2682-5p was displayed in RA synovial tissues (Figure 5H). Moreover, miR-2682-5p level was negatively correlated with either ZFAS1 or ADAMTS9 in RA synovial tissues (Figure 5I,J). Collectively, ZFAS1 was a sponge of miR-2682-5p to regulate ADAMTS9 in RA synovial tissues.

### ZFAS1 exerted its function via miR-2682-5p/ADAMTS9 axis in FLS-RA

**Figure 6:**
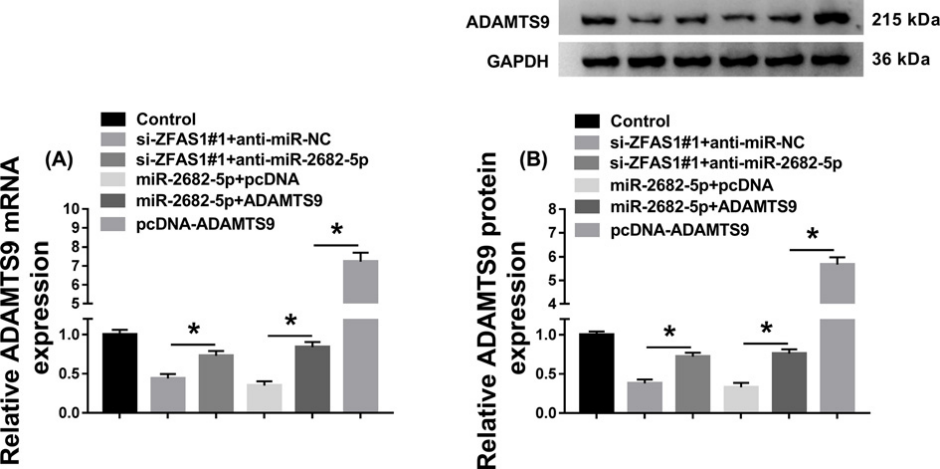
ZFAS1/miR-2682-5p axis modulated the level of ADAMTS9 (**A** and** B**) Si-ZFAS1#1+anti-miR-NC, si-ZFAS1#1+anti-miR-2682-5p, miR-2682-5p+pcDNA, miR-2682-5p+ADAMTS9, or pcDNA-ADAMTS9 was introduced into FLS-RA, respectively, and the level of ADAMTS9 was identified at the aspects of mRNA and protein expression; *P<0.05.

**Figure 7:**
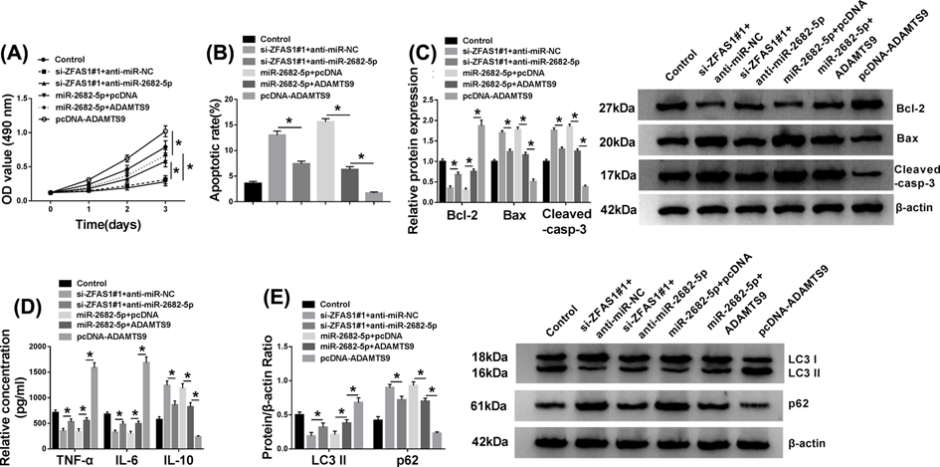
ZFAS1 exerted its function via miR-2682-5p/ADAMTS9 axis in FLS-RA FLS-RA were transfected with si-ZFAS1#1+anti-miR-NC, si-ZFAS1#1+anti-miR-2682-5p, miR-2682-5p+pcDNA, miR-2682-5p+ADAMTS9, or pcDNA-ADAMTS9, severally. (**A**) MTT assay was employed to evaluate the proliferation of FLS-RA. (**B**) The alteration of cell apoptosis was shown. (**C**) Western blot assay was administrated to reckon the levels of Bcl-2, Bax, and cleaved-casp-3 *in vitro*. (**D**) ELISA was performed to determine the changes of TNF-α, IL-6 and IL-10 secretions. (**E**) The levels of LC3II and p62 were examined by Western blot; *P<0.05.

As mentioned above, we systemically investigated the functional mechanism between miR-2682-5p, ZFAS1, and ADAMTS9 subsequently. First, RA synoviocytes were transfected with si-ZFAS1#1+anti-miR-NC, si-ZFAS1#1+anti-miR-2682-5p, miR-2682-5p+pcDNA, miR-2682-5p+ADAMTS9, or pcDNA-ADAMTS9, respectively. As described in Figure 6A,B, miR-2682-5p inhibitor could restore the repressive impact of ZFAS1 silencing on the mRNA and protein levels of ADAMTS9 in FLS-RA. Also, reintroduction of ADAMTS9 relieved the inhibitory effect of miR-2682-5p transfection on the level of ADAMTS9 at the aspects of mRNA and protein expression in RA synoviocytes, and the introduction of pcDNA-ADAMTS9 only could be enhanced the mRNA level and protein level of ADAMTS9 in RA synoviocytes. Then, MTT analysis disclosed that the reduction effect of ZFAS1 detetion on cell proliferation was notably regained via miR-2682-5p inhibition *in vitro*. Similarly, overexpression of miR-2682-5p distinctly declined the proliferation of FLS-RA, while increase in ADAMTS9 significantly abrogated this effect. Meanwhile, enhanced proliferation due to the up-regulation of ADAMTS9 was viewed *in vitro* (Figure 7A). Moreover, ZFAS1 deficiency resulted in a significant augment in apoptotic rate in FLS-RA, which was evidently eliminated by regaining of miR-2682-5p inhibitor. However, miR-2682-5p-mediated boost in cell apoptosis was reverted after simultaneous transfection with ADAMTS9. And the overexpression of ADAMTS9 reduced the apoptosis rate of FLS-RA (Figure 7B). Also, the changes of Bcl-2, Bax, and Cleaved-casp-3 levels illustrated the conclusion of cell apoptosis in FLS-RA (Figure 7C). At the same time, silencing of ZFAS1 led to the curb of inflammatory response, which was markedly overturned by reintroducing of anti-miR-2682-5p. Nevertheless, miR-2682-5p-regulated repression in inflammation was reverted after co-transfection with ADAMTS9 in FLS-RA, and the transfection of pcDNA-ADAMTS9 alone facilitated the inflammation (Figure 7D). Furthermore, a similar regulatory mechanism was observed in cell autophagy in comparison with the modulation in inflammatory response in FLS-RA (Figure 7E). Altogether, ZFAS1 might function its oncogenic role via miR-2682-5p/ADAMTS9 axis in RA

## Discussion

Until now, growing evidence has displayed that lncRNAs worked as various roles in diverse biological regulation, including cell growth, apoptosis, autophagy and inflammatory response [27]. It has been demonstrated that the ectopic expression of lncRNAs is extremely related to multiple human diseases, implying that lncRNAs may serve as the novel biomarkers in the diagnosis, therapy, and prognosis in diseases, including inflammatory diseases [28]. One reports disclosed that ZFAS1 could improve the migration and invasion in FLS-RA by inactivating miR-27a [29], and this conclusion prompted that ZFAS1 might be involved in the pathogenesis and development of RA, and it is of great significant to discuss the influence of ZFAS1 on RA and accurately describe the regulatory mechanism. Of which FLS-RA was proved to be the master effector cells in RA [30], all the assays were carried out using the separated FLS-RA in the present study.

In accordance with previous introductions, ZFAS1 serves as an oncogenic lncRNA in several human carcinomas. For instance, the deficiency of ZFAS1 could weaken the aggression of gastric cancer cells by suppressing Wnt/β-catenin signaling pathway [31]. ZFAS1 also facilitated the progression of glioma by activating Notch pathway [32]. In addition, the high expression of ZFAS1 in osteosarcoma was reported to function as a tumorigenic lncRNA, indicating the promotion of cell growth and metastasis [33]. That was to say, ZFAS1 was significantly connected with human cancer. In the present study, we focused on the role of ZFAS1 in modifying the progression of OA. First, an obvious up-regulation of ZFAS1 in FLS-RA was observed, and ZFAS1 detetion could effectively retard cell proliferation and accelerate apoptosis *in vitro*. Apoptosis is deemed as a critical mechanism that modifies tissue composition and homeostasis [34]. The ectopic change of cell apoptosis has been discovered in residential synoviocytes and inflammatorycells [35,36]. Collectively, we guessed that ZFAS1 might be specially connected with the inflammatory response, and the alterations of IL-6, IL-10, and TNF-α level manifested the hypothesis. Besides, the regulatory tendency of cell autophagy was determined, ZFAS1 detetion could suppress the autophagy of FLS-RA. Next, the present study searched for the functional targets of ZFAS1 by using starBase v3.0 software.

Currently, the interrelation between lncRNAs and miRNAs has attracted much attention. Several literatures have been suggested to be related to ectopic expression of lncRNAs and the involved miRNAs with dysregulation in various diseases [37]. For example, gastric adenocarcinoma predictive long intergenic non-coding (GAPLINC) could expedite the cancer-like behaviors in FLS-RA in RA patients by serving as the sponge of miRNAs [38]. Furthermore, the role of mRNAs in modulating inflammatory response has been recorded [39]. In the present investigation, we found that there were complementary sites between ZFAS1 and miR-2682-5p, and the biological influence of ZFAS1 on cell behaviors and inflammation was overturned after co-transfection with anti-miR-2682-5p in FLS-RA. Coupled with the results of dual-luciferase reporter assay, we concluded that miR-2682-5p was a target of ZFAS1. In regard to miR-2682-5p, few researches were reported before, only one studies focused on the role of miR-2682-3p on the progression of osteosarcoma [18]. The biological function of miR-2682-5p, a newly identified miRNA, might provide a novel insight into the diagnosis and treatment of human RA in the clinic.

According to the prediction of miRDB software, ADAMTS9 was regarded as the possible target of miR-2682-5p, and this guess was validated by subsequent assays. ADAMTS9, it has been proved to be the target of miR-338-5p, thereby participating in the proliferation and invasion of FLS-RA [26]. However, ADAMTS9 acts as the tumor-suppressive gene in several human cancer cells, such as the process of tumor formation was suppressed in esophageal and nasopharyngeal carcinoma [40]. Namely, ADAMTS9 assumed the different monitor and regulator in diverse diseases.

Taken together, ZFAS1 and ADAMTS9 with high levels and miR-2682-5p with low level took part in the progression of RA. Knockdown of ZFAS1 could reduce cell proliferation, autophagy, inflammatory response, and elevate cell apoptosis in FLS-RA. We first demonstrated that ZFAS1 functioned as a major modulator through miR-2682-5p/ADAMTS9 axis in FLS-RA. Nevertheless, the evidence is not sufficient to expound that ZFAS1 serves as an effective molecular marker in RA treatment, more investigations are required to understand the potential role of ZFAS1 in OA development.

## Highlights

1. ZFAS1 and ADAMTS9 were up-regulated, while miR-2682-5p was low-expressed in RA and FLS-RA.
2. Knockdown of ZFAS1 could sharply curb cell proliferation, inflammatory response, autophagy, and promote apoptosis in FLS-RA.
3. ZFAS1 functioned its role via miR-2682-5p/ADAMTS9 axis in RA.

## Abbreviations

ELISA: enzyme-linked immunosorbent assay
MTT: 3-(4,5-dimethylthiazol-2-yl)-2,5-diphenyltetrazolium bromide
qRT-PCR: quantitative real-time polymerase chain reaction
RA: rheumatoid arthritis
TNF: tumor necrosis factor
